# Early Child Development Assessments and Their Associations with Long-Term Academic and Economic Outcomes: A Systematic Review

**DOI:** 10.3390/ijerph18041538

**Published:** 2021-02-05

**Authors:** Leah N. Isquith-Dicker, Andrew Kwist, Danae Black, Stephen E. Hawes, Jennifer Slyker, Sharon Bergquist, Susanne P. Martin-Herz

**Affiliations:** 1 Department of Global Health, University of Washington START Center, Seattle, WA 98195, USA; leahiuw@gmail.com (L.N.I.-D.); andrew.kwist@gmail.com (A.K.); danaesb@uw.edu (D.B.); hawes@uw.edu (S.E.H.); jslyker@uw.edu (J.S.); 2 Department of Anthropology, School of Public Health, University of Washingto, Seattle, WA 98195, USA; 3 Department of Epidemiology, School of Public Health, University of Washington, Seattle, WA 98195, USA; 4 Bill & Melinda Gates Foundation, 500 5th Ave N, Seattle, WA 98109, USA; sharon.bergquist@gatesventures.com; 5 Department of Pediatrics, University of California San Francisco, 1825 Fourth St., 6th Floor, UCSF Box 4054, San Francisco, CA 94143, USA

**Keywords:** child development, functioning, outcomes, education, measurement, child development assessment, academic achievement, educational attainment, wealth/socioeconomic status

## Abstract

Developmental screening instruments were designed as diagnostic tools, but there is growing interest in understanding whether select tools can also be used systematically in research to examine intervention impacts on long-term outcomes. As such, this systematic review aims to examine associations between child development assessment tools and educational attainment, academic achievement, or wealth. We included studies identified in PubMed, PsycINFO, and Educational Resources Information Center if they reported an association between at least one tool from a pre-established list and one outcome of interest after age 10. Of 597 studies identified, 11 met inclusion criteria; three examined educational attainment as the outcome of interest, six examined academic achievement, one wealth, and one both educational attainment and wealth. Intelligence tests were utilized in five of the included studies, neuropsychological/executive function or behavior tools were used in five, and one study used tools across the domains. High-quality studies were identified across all three of the domains, but educational attainment and wealth had the greatest proportion of high-quality studies, as compared to academic achievement. Our review demonstrates the potential for certain child development assessment tools to adequately assess long-term outcomes of interest, but additional prospective studies using validated, culturally appropriate tools are needed. PROSPERO registration number: CRD42018092292.

## 1. Introduction

Recent findings from the *Lancet* series on early childhood development estimate that over 250 million children under five years of age in low-and middle-income countries are at risk of not achieving their full developmental potential [1]. Children in low- and middle-income countries may face a variety of adversities, including recurrent illness, malnutrition, and trauma, with long-term consequences for their health, productivity, and overall well-being [2]. It has been previously estimated that children not reaching their full developmental potential results in an average adult annual income deficit of 19.8% [3]. In addition to income loss, developmental delays have also been associated with considerable academic underachievement [4,5,6]. These factors have led to increased interest in child development assessment tools that are adequately associated with long-term academic, economic, and human capital outcomes from a young age. However, existing child development assessment tools were generally designed to assess risk or developmental status at the time of testing [7,8,9,10]. Limited research focused on this topic suggests that available tools may not have strong predictive potential for later outcomes [1,4], highlighting the need for additional investigation to determine if specific tools or developmental domains may be useful for both prediction of outcome without intervention or in evaluation of intervention programs.

This systematic review was conducted to identify child development assessment tools that are associated with the long-term outcomes of educational attainment, academic achievement, and wealth. Given that an assessment of study quality is also included in this review, this synthesis provides information to aid researchers in the selection and prioritization of tools or domains for further research in this area.

## 2. Materials and Methods

### 2.1. Data Sources

A literature search was conducted in PubMed, PsycINFO, and Educational Resources Information Center (ERIC) on March 30, 2018. Publication date was restricted to manuscripts published in 1990 or later, due to differences in assessment tools and publication standards in comparison to more recent literature (Document S1). A search template was created that was applied to all 3 databases with minimal tailoring (Document S2). The search yielded 597 unique results that were exported into the Covidence (Melbourne, Australia) systematic review software [11]. This systematic review was conducted in accordance with PRISMA (Preferred Reporting Items for Systematic Reviews and Meta-Analyses) guidelines and preregistered at PROSPERO: CRD42018092292. 

### 2.2. Development Tool Selection

The search template contained a list of 104 child development assessment tools (Table S1). An initial list of 1398 tools was generated from a search of the PsycTEST database, a repository of assessment tools indexed from articles in peer-reviewed journals and books by experts at the American Psychological Association [12]. The PsycTEST search utilized the search terms, “child development, executive function, school, education, academic, readiness, reading, literacy” and excluded the terms “sexual behaviors and divorce.” As many tools were not relevant to study objectives, one of the authors (SMH) manually filtered the list and added additional relevant assessment tools absent from PsycTEST database to assemble the final list. Tools were retained in the final list if they measured at least one of the five most common domains of development (gross motor, fine motor, language, cognitive, and social emotional), or examined reading/pre-reading skills or executive function. Tools were required to show some indication of psychometric properties, since it was felt that at least some evidence of reliability and validity were prerequisites to adequate association between the tools and future educational and economic outcomes, even if this was obtained from cross-sectional as opposed to longitudinal research. Tools were excluded if they examined only sublevels of developmental domains, were specific to second language learning, were designed explicitly to evaluate gender differences, were specific to health or mental health diagnoses, were specific to a particular study context, examined child or caregiver attitudes toward a developmental domain or skill set rather than the child’s development (e.g., child reading attitude), or focused specifically on theory of the mind, math achievement, or home literacy environment.

### 2.3. Study Inclusion and Exclusion Criteria

Studies were included in this review on the basis of the following criteria: (1) the study was conducted using an experimental or observational design; (2) the study used at least one of the assessment tools in the list with children under the age of 18; (3) the study reported at least one outcome related to educational attainment, academic achievement, or wealth and assessed this outcome after the age of 10 years; educational attainment was defined by the highest-grade level completed at the time of data collection, while academic achievement was defined as performance in one or more subject areas; (4) articles were published in English or French; French-language articles were included in an attempt to ensure inclusion of articles that were conducted in Francophone Africa.

Cross-sectional studies were excluded because the study objective was to identify tools that were associated with long-term academic and socioeconomic outcomes. Studies conducted with children who were hospitalized or had severe neurologic injuries, genetic conditions, or autism spectrum disorder were excluded, because the use of a tool in such a sample could not easily be generalizable to the larger pediatric population. The age of 10 was chosen as a lower bound for long-term outcomes due to concern that children in some low-and middle-income countries may terminate formal schooling near this age. There were no exclusion criteria related to the length of time between tool assessment and outcome evaluation or the specific geographic location. Detailed inclusion and exclusion criteria can be found in the study’s PROPSERO preregistration.

### 2.4. Research Processes

**Figure 1:**
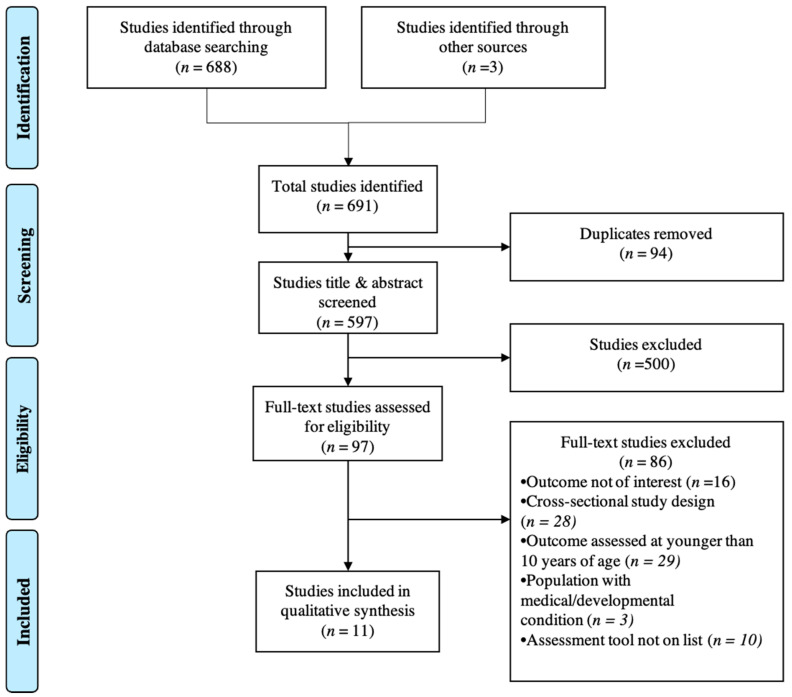
A Preferred Reporting Items for Systematic Reviews and Meta-Analyses (PRISMA) flowchart of the selection process of published studies.

Title and abstract screening were completed for all identified studies independently by two reviewers (LNID and AK), and discordance was resolved by a third reviewer (JS). Full text screening was also completed by two reviewers (LNID and AK), with discordance resolved by a third reviewer (DB). For studies excluded by full-text review, reviewers selected one primary reason for exclusion, and discordance in study exclusion rationale was resolved by a third reviewer (JS). The exclusion hierarchy was as follows, in order of priority: outcome not of interest, cross-sectional study design, outcome assessed at younger than 10 years of age, population with medical/developmental condition, assessment tool not on list. A PRISMA flowchart is provided in Figure 1.

In addition to this structured search, targeted searching was conducted in two ways. First, publications identified from a list of cohort studies recommended by subject matter experts (SMH and SB) were evaluated for their adherence to study inclusion criteria [13,14,15,16]. Second, an additional search using cohort studies identified during the initial stages of the title and abstract screening process was undertaken, and three potentially relevant studies were added to the total pool for title and abstract screening (additional studies identified through other sources in Figure 1). These additional steps were taken in an effort to broaden the scope of the review in the event that the search terms missed relevant studies.

### 2.5. Data Abstraction

The following information was abstracted from the articles identified in the full-text review: author, publication year, sample size, sampling method, time between assessment tool and outcome, assessment tool(s) used, age at which assessment tool was used, outcome(s), age at which outcome was evaluated, and measure(s) of association. For studies that used an assessment tool at multiple time points without separate effect measures, we only reported the greater duration from child development assessment to outcome (e.g., for tool applied at age 11 and age 16 and outcome measured at age 19, we only reported association between age 11 to age 19). In some studies, more than one assessment tool was used, including some child development assessment tools that were not on our pre-specified list. In these cases, only the associations between the tools on the list and our outcomes of interest were reported. Effect measures are presented adjusted for standard sociodemographic factors (i.e., parent education, sex, and wealth) unless otherwise stated. Regression coefficients and odds ratios are presented with statistical significance, and confidence intervals when measures of statistical significance were unavailable.

### 2.6. Data Synthesis

We used a narrative approach to data synthesis, which is the preferred method when empirical approaches and variables are highly varied across studies [17]. Tools were then categorized by the domain or construct area they assessed. This classification was initially made by a developmental-behavioral pediatrician (SMH) and then independently by 3 volunteer neuropsychologists. We used outcome, tool domain, and a study quality assessment as the classification schemes for synthesizing data.

### 2.7. Quality Assessment

A quality assessment was conducted to determine the rigor of study methods and relevance of the effect measures reported, in the context of the objectives of our study. For the quality assessment, five criteria were evaluated by the two authors (DB and LID) who completed the data extraction. These criteria were adapted from the Cochrane Collaboration Risk of Bias Tool, usually applied to evaluations of quantitative evidence from systematic reviews [18]. The authors of this tool recommended the assessment of five specific categories of bias and one category for other types of bias as needed in accordance with study objectives. Detail on bias categories is provided below; other categories were not considered given the sufficient coverage provided by those applied. The authors evaluated the criteria on these categories independently by maintaining separate data sheets that were shared only when evaluations were complete. Discordance was discussed and resolved by a third reviewer (JS). Each criterion is defined below and was assigned a designation of, low, high, or unclear quality with point values of −1, 1, and 0, respectively. Criteria were weighted equally because each category of bias was deemed of equal importance to study quality. An overall assessment of study quality was ascertained by the sum of the point values, termed the "cumulative quality assessment", which was used to categorize studies as of high, low, or neutral quality. The unclear designation was assigned when information regarding a criterion were not provided in the article itself and was not possible to ascertain from supplemental materials or earlier publications describing study methods. Quality assessment criteria were as follows:

(1) Selection: Evaluated how participants were selected for inclusion in the study. A study with a random sample or an attempted census was designated high quality and a convenience sample was designated as low quality.
(2) Sample size: Examined the adequacy of the sample size. A total sample size of >100 participants was designated high quality for the current review, as smaller studies may have very limited statistical power.
(3) Attrition: Assessed loss to follow up, withdrawal, or exclusion from analysis. Studies with a rate of attrition of < 25% were designated high quality.
(4) Duration: Evaluated the length of time between child development assessment and outcome. Studies with durations >5 years were considered to be high quality.
(5) Outcome reporting: Evaluated the extent to which authors provided information on how outcomes were measured. Standardized tests, registries, or assessment tools and those reported by teachers or trained experts (e.g., psychologist) were designated high quality, and self-report (child or parent) was designated low quality.
(6) Cumulative quality assessment: Sum of point values for criterion 1–5.

## 3. Results

### 3.1. Overview of Studies

Of 597 unique articles identified, 500 were excluded during the title and abstract screening phase, largely due to being cross-sectional in nature, not assessing an outcome of interest, or having the outcome of interest but assessing it prior to 10 years of age. During this phase, none of the publications identified by subject matter experts for targeted searching met criteria for full-text review. Of the 97 articles included in the full text review phase, 11 met study criteria for inclusion (Table S2); the number of articles excluded for each criterion is documented in Figure 1. All included studies were observational cohort studies, with a follow-up duration range from 2 years to greater than 20 years. Six studies were sampled from a school setting [19,20,21,22,23,24], two from birth cohorts [25,26], one via an adoption agency [27], and two through larger population-based studies [28,29]. Sample sizes varied widely, with smaller cohorts (<50) among special populations (e.g., low-income families) and larger population-based cohorts (>6000).

### 3.2. Assessment Tool Domains of Studies Included for Review

**Figure 2:**
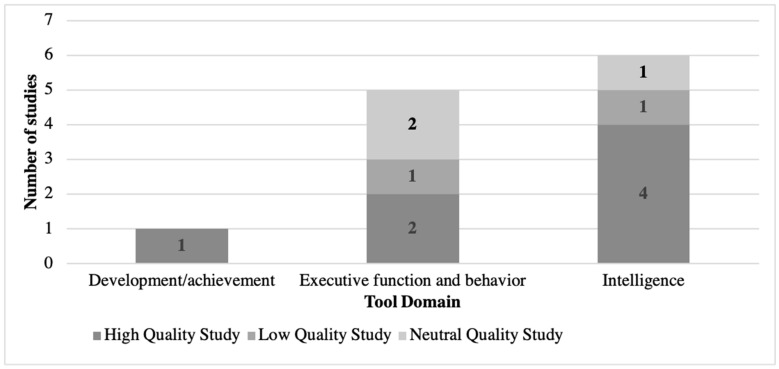
Included studies by domain of child development assessment tool. The total number of studies listed exceeds the number of studies included in this review because [27] included 2 child development assessment tools of interest in 2 domains (Development/achievement and Intelligence). A detailed explanation for the high/neutral/low quality designation is provided in Section 3.4 below.

The child development assessment tools utilized in the 11 included studies were classified into three categories: neuropsychological/executive function and behavior, intelligence, and general development and achievement (Table 1). Figure 2 displays the distribution of studies by the domain of the tool used. Neuropsychological/executive function and behavior tools were employed in five studies; intelligence tests in five studies, and multiple tools from both the general development/achievement and intelligence domains were used in one study (see Figure 2 notes for details). Studies that analyzed multiple tools independently were counted separately for each tool.

### 3.3. Outcomes

**Figure 3:**
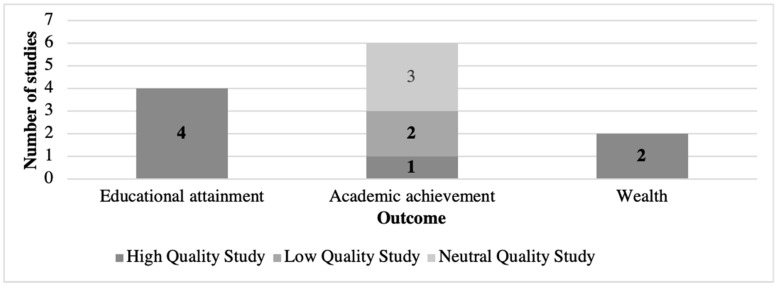
Included studies by outcome of interest. NOTES: The total number of studies listed exceeds the number of studies included in this review because [25] assessed both educational attainment and wealth. A detailed explanation for the high/neutral/low quality designation is provided in Section 3.4 below.

The association between a child development assessment tool of interest and educational attainment was measured by four of the 11 selected studies, as shown in Figure 3 [25,27,28,29]. Educational attainment was determined either by self-report of the number of school years completed or national registries that included school completion information. Six studies reported associations between a tool of interest and academic achievement [19,20,21,22,23,24]. There was more heterogeneity in the measurement of academic achievement, including standardized tests that were named (e.g., Iowa Test of Basic Skills, Metropolitan Achievement Test, and General Certificate of Secondary Education) or unnamed, as well as school grade point averages, either from school records or by self-report. Only two studies assessed outcomes related to wealth, income, or socioeconomic status [25,26].

### 3.4. Quality Assessment

Table 2 displays the results of the quality assessment, which identified two low quality studies, four neutral quality studies, and five high quality studies. The low-quality studies were published in 1995 and 2017 and represented small samples of children attending a residential school in Canada (N = 20) and an elementary school in Switzerland (N = 103), respectively [20,21]. These studies reported non-significant effect estimates for outcomes and had short duration of follow-up (≤3 years). The high-quality studies were published between 2001 and 2014, included large cohorts (>1000) from New Zealand, Norway, and The Netherlands, and a smaller cohort from the United States [22,25,26,28,29]. The effect measures for the high-quality studies were almost all significant (except one effect measure from a study that used the Youth Self Report & Child Behavior Checklist [29] and had greater length of follow up (range 5 to 29 years). The five high quality studies are summarized below:

- Moffitt, 2011 [26]

A prospective cohort study from the participants in the Dunedin Multidisciplinary Health and Development Study Cohort in New Zealand assessed childhood self-control, socioeconomic factors, and IQ using the Wechsler Intelligence Scales for Children, Revised (WISC-R; repeat measures at ages 3, 5, 7, 9, and 11), and the association with wealth at age 32. Statistical models included adjustment for socioeconomic factors and fixed-effects modeling applied to dizygotic same-gender twins to compare outcomes of siblings with differential self-control levels and thus isolate the effect of self-control. The study found that the intelligence assessment was significantly associated with four measures of wealth: socioeconomic status, income, financial planfulness, and financial issues (regression estimates −0.400, −0.291, −0.160, and 0.029, respectively; all *p* < 0.05).

- Sagatun, 2014 [28]

A retrospective cohort study that utilized data from a Norwegian registry to assess the association between the Strengths and Difficulties Questionnaire administered to 15- to 16-year-olds and academic attainment as recorded in the national registry of school completion at age 20–21. Statistical models included adjustments for children’s ethnic background, county of residence, parents’ education, income, and marital status. The study found that this tool was significantly associated with odds of non-completion of school (ORs 1.11–1.48, all *p* < 0.001).

- Lamp, 2001 [22]

A prospective cohort study among families enrolled in the Head Start Program in the United States assessed intelligence using the Stanford Binet Intelligence scale at age 4 and its correlation with academic achievement at ages 5 to 10 years, measured by the Metropolitan Achievement Test. No information regarding the factors used for adjustment in statistical models was provided. The study found that intelligence as measured by this tool was significantly correlated with academic achievement (correlation coefficients 0.39–0.62, all *p* < 0.01).

- Fergusson, 2005 [25]

A retrospective cohort study involving participants from the Christchurch Child Development Study in New Zealand assessed intelligence using the WISC-R in 8 to 9-year-olds and analyzed its association with wealth, educational outcomes, and obtaining a university degree between the ages of 18 and 25 years. Statistical models included adjustment for a series of covariate factors including measures of childhood social and family disadvantage and behavior. The study found that intelligence was significantly associated with gross income (regression coefficient 1.595, *p* < 0.05) and gaining school or university qualifications (regression coefficients 0.67–0.82, *p* < 0.01).

- Veldman, 2014 [29]

A prospective cohort study to determine likelihood of educational attainment (measured by number of years of schooling completed) by age 19, using data from the Tracking Adolescent’s Individual Lives Survey in The Netherlands, assessed 11 year-olds using the Child Behavior Checklist and its Youth Self Report. Statistical models included adjustment for children’s sex, age, IQ, parental educational status, and physical health status. The study found that externalizing, internalizing, and attention problems, as assessed by these combined tools, were associated with higher odds of low (primary, lower vocational and lower secondary education) vs. medium (intermediate vocational and intermediate secondary) educational attainment at age 19 (OR 1.25–1.78; statistical significance varied—see Table S3 for details).

## 4. Discussion

### 4.1. Overview of Key Findings

This study sought to examine the evidence base for the association between child development assessment tools and longer-term outcome. After applying a rigorous set of inclusion criteria on 597 studies identified from our initial search, we retained 11 observational cohort studies in this systematic review that investigated the association between a child development assessment tool of interest and a long-term outcome of interest. Although the studies were distributed across all three outcomes of interest, and three development tool domains, the majority of these studies investigated the outcome of academic achievement and used intelligence or neuropsychological/executive function and behavioral tools as predictors. Five of the eleven studies were determined to be high quality and reported measures of association that were almost all significant; given that these studies had at least 100 participants, and a minimum of 5 years duration of follow-up, these would have more statistical power to show a significant effect size. These findings suggest that child development assessment tools across a range of development domains may have predictive potential for various types of outcomes later in life, but several limitations of the available literature and limitations of our study suggest that further research is needed as described below.

### 4.2. Limitations of the Available Literature

The evidence base supporting the ability of child development assessment tools to predict long-term outcomes remains limited to remarkably few studies, with a need for more high-quality studies that are adequately powered and have follow-up sufficient to reveal associations with adult-life outcomes. Figure 2 and Figure 3 illustrate that there are high quality studies distributed across the three outcomes of interest and all three assessment tool domains. However, the included studies were heterogeneous with respect to study design, assessment tools, outcome measures, and statistical models. This heterogeneity precludes direct comparison, even between studies that used the same tool (e.g., WISC-R) to determine whether these associations are repeatable, and the effect sizes are consistent across populations. Our quality assessment suggests that issues related to attrition remain a challenge in longitudinal studies; continuing to engage and track study participants over decades is a common challenge in longitudinal studies, so this finding is not all together surprising. However, it is notable that two studies did not clearly describe attrition, which threatens both evaluation of sample size and effect measures [20,24].

All included studies in this review were observational cohort studies, which are susceptible to several limitations. Cohort studies are prone to differential loss to follow-up of participants with medical or financial challenges, which can bias findings. While many studies accounted for confounding with adjusted effect estimates, additional sources of residual confounding likely remained, including family and community contextual factors, the impact of developmental interventions, and children’s physical health. Longitudinal studies that document and control for these contextual factors are needed.

Additionally, the use of multiple or composite assessment tools was framed as a “best fit” approach by some authors. However, the utilization of multiple predictors can diminish the statistical validity of significant results due to the increased probability of a significant result due solely to chance, given the large number of hypothesis tests. A priori assertions grounded in theoretical rationale for the utility of composite or multiple domain assessment tools can help to mitigate this issue and provide better evidence as to whether composite assessments improve prediction of outcomes; alternatively, the assessment of predictors separately would help to isolate the effect of individual tools.

Finally, the generalizability of findings from this review is limited by the fact that all of the studies took place in high-income countries among relatively homogenous racial and ethnic groups. Few of the tools assessed in this review have been validated for use in African, Asian, and South American populations. The absence of studies from low-and middle-income countries may be a reflection of the small number of tools validated for use in these populations, and limits generalizability of findings to populations from low-income countries, and populations with high rates of malnutrition or limited access to education.

### 4.3. Limitations of Present Study

There are several limitations to this review. First, the study was designed with a specific purpose to identify developmental assessment tools that predict long term outcomes related to academic and economic potential of individuals and communities and did not include research assessing other long-term outcomes with high relevance for health and quality of life. Despite efforts to be comprehensive in its inclusion of tools by completing a broad search of the PsycTESTS database and reviewing almost 1400 tools, some studies were excluded at full-text review because they did not include an assessment tool from the original search list (e.g., a study that examined educational attainment among three large cohorts from Finland, the UK, and the Philippines and found significant positive associations between cognitive development scores at early ages and attainment in adulthood [30]). Despite a thorough search of three robust databases, there is likely additional relevant research that was not captured. In particular, grey literature, such as non-peer reviewed organizational reports, and economics literature (e.g., EconLit database) were not considered and may be a source of additional information regarding the socioeconomic outcome of interest. Additionally, only English and French literature was reviewed due to the linguistic capacity of the research team, and thus there may be additional literature in other languages that may be particularly relevant to address the issue mentioned above related to generalizability of findings to the low-and middle-income country context.

Next, this review was completed in 2018; to remediate the concern of additional published literature not being reflected in this review, in January 2021 we conducted post-hoc abstract screening of articles published in 2018–2021 in all three databases (PubMed, Educational Resources Information Center (ERIC), and PsycINFO), using the same search terms. Of 158 results across the three databases, five articles passed abstract screening and were full-text reviewed, and only two additional studies met inclusion criteria [31,32]. First, Samuels et al., 2019 found that the Behavior Rating Inventory of Executive Function (BRIEF) and BRIEF Self-Report (BRIEF-SR) were significantly associated with the upcoming cumulative grade point average in a diverse population of 259 New York middle and high school students, independent of gender, free/reduced lunch, and special education status [31]. However, it is unclear whether this instrument predicts longer-term academic performance because the time interval between tool assessment and outcome assessment was notably short. Second, Kosik et al., 2018 found in a U.S based birth cohort that the WISC at age seven was significantly associated with educational attainment, employment, and wealth in adulthood [32]. Despite the identification of these two additional studies, of which likely only Kosik et al., 2018 would be considered high-quality, we are confident that the findings reported in our main review remain relevant and continue to fill a needed gap in the literature. These studies’ findings do not conflict with findings of the five high-quality studies in the main review, and in fact only further support our review’s overall conclusions.

Finally, all of the high-quality studies reviewed reported positive associations, suggesting publication bias and potential underreporting of null findings. Coupled with the small sample sizes and shorter follow-up of the low and neutral quality studies reviewed, additional research is needed to support the associations identified between tools and outcomes studied herein.

### 4.4. Recommendations for Future Research

Additional research evaluating regionally-validated tools, conducted in large and diverse study populations with adequate follow-up, including low-and middle-income countries, are needed to understand whether these tools can be used to predict long term outcomes and assess the impact of interventions. Existing data from large cohort studies in these low-and middle-income countries, either ongoing or already completed, could also be leveraged to contribute to this field of work. Many of the tools evaluated in our review were proprietary, and there is growing interest in developing tools that are valid across multiple populations and that can be administered by medical staff or community health workers [33]. Additionally, to address the limitation of the inability to capture all potentially relevant development tools of interest, researchers conducting future research on this topic could consider not restricting their search to specific tools, but instead develop a detailed search string on keywords related developmental domains.

## 5. Conclusions

Our review identified 11 studies investigating associations between early childhood assessment tools and long-term economic and academic outcomes of interest. Five of these studies were determined to be high-quality and reported mostly statistically significant associations, suggesting that certain child development assessment tools are associated with the long-term outcomes of interest. Given that child development assessment tools were designed to identify children with developmental delay at the time of assessment, our study addresses a key need to characterize the potential for these tools to be sensitive to intervention effects and to potentially predict longer-term outcomes. The high-quality literature reviewed was primarily conducted in high-resource contexts and was relatively sparse; as such, additional prospective studies, engaging large, diverse populations in both high-income and low-and middle-income countries are needed to adequately address remaining gaps in this evidence base.

## Figures and Tables

**Table 1:** Child development assessment tools utilized in included studies, by domain.

Domain	Tools
Neuropsychological/ executive function and behavior	**CBCL—Child Behavior Checklist**
	RINT—Reitan-Indiana Neuropsychological Battery for Children
	SDQ—Strengths and Difficulties Questionnaire
	SMFQ—Short Mood and Feelings Questionnaire
	YSR—Youth Self Report of the Child Behavior Checklist
	CAAS—Children’s Attention and Adjustment Survey
Intelligence	Stanford-Binet FE (Fourth Edition)
	Stanford-Binet: LM (Form LM)
	WISC-R—Weschler Intelligence Scale for Children-Revised
	WISC Verbal IQ
	WISC Performance IQ
Development/achievement	PIAT—Peabody Individual Achievement Test-Reading subscale
	PPVT-R—Peabody Picture Vocabulary Test-Revised

**Table 2:** Study quality assessment.

Author, Year	Selection	Attrition	Outcome Reporting	SAMPLE SIZE	Duration	Cumulative Assessment
Moffitt, 2011 [26]	High	High	High	High	High	5 (High)
Sagatun, 2014 [28]	High	High	High	High	High	5 (High)
Lamp, 2001 [22]	High	High	High	Low	High	3 (High)
Fergusson, 2005 [25]	High	High	Low	High	High	3 (High)
Veldman, 2014 [29]	High	High	Low	High	High	3 (High)
Clarren, 1993 [19]	Low	Low	High	High	High	1 (Neutral)
Rothon, 2009 [23]	High	Low	High	High	Low	1 (Neutral)
Samuels, 2016 [24]	Unclear	Unclear	High	Unclear	Low	0 (Neutral)
McClelland, 2013 [27]	Low	Low	Low	High	High	−1 (Neutral)
Richards, 1995 [20]	Low	Unclear	High	Low	Low	−2 (Low)
Gygi, 2017 [21]	Unclear	Low	Low	Low	Low	−4 (Low)

Each criterion was evaluated with the following numerical values: high quality = 1; low quality = −1, unclear quality = 0. Each study could receive up to a cumulative assessment value of 5. Studies with values > 1 were designated high quality studies, values of 1, 0 and −1 neutral quality, and < −1 low quality studies. See Section 2.7 (Materials and Methods: Quality Assessment) for additional detail.

## Data Availability

Not Applicable.

## Supplementary Materials

The following are available online at https://www.mdpi.com/1660-4601/18/4/1538/s1, Document S1, Document S2: PubMed, PyscINFO, and ERIC database search strings, Table S1: Child development assessment tools included in search string, Table S2. Details of studies included in the review.
