# Supplementary material for: Early Child Development Assessments and Their Associations with Long-Term Academic and Economic Outcomes: A Systematic Review
Source: Int J Environ Res Public Health. 2021 Feb 5;18(4):1538. doi: 10.3390/ijerph18041538 (PMC7915620; doi:10.3390/ijerph18041538)
Supplement: Supplementary file 1 [file ijerph-18-01538-s001.pdf]

SUPPLEMENTAL DOCUMENT 1.

The decision to consider only manuscripts published in 1990 or later was made after consideration of available early childhood development tools and research methodology.

Three prominent developmental assessment tools in use currently across the globe were either developed or updated and re-normed from their first version around this time. This includes the Bayley Scales of Infant Development, which was initially developed in 1969 but updated and re-normed in 1993 (Bayley II), the Mullen Scales of Early Learning, which was published in 1985, and the Griffiths Mental Development Scales, which were originally developed in 1970 but updated and validated in 1996. It was also important to the goal of the study that early childhood development assessment tools included were still in use and could be validly implemented if found to show longitudinal predictive ability.

The past 3 decades have also seen a significant improvement in research study quality, including larger sample sizes, more sophisticated modeling techniques, improved recognition of and adherence to research ethics, and stricter publication requirements. Although somewhat arbitrary, together these factors lead the team to cut the search at the January 1 1990.

SUPPLEMENTAL DOCUMENT 2. PubMed, PyscINFO, and ERIC database search strings

PUBMED SEARCH

(“Swedish Communicative Development Inventory III”[tw] OR “Kindergarten Student Entrance Profile-Adapted Version”[tw] OR “Environmental Symbols Instrument”[tw] OR “Representational Knowledge Assessment”[tw] OR “SEEK Observational Instrument”[tw] or “Nonword Reading Task”[tw] OR “Kindergarten Readiness Checklist-Chinese Version”[tw] OR “Object-based Pattern Reasoning Assessment”[tw] OR “Australian English Communicative Development Inventory”[tw] OR “Child Performance Skills Questionnaire-Adapted”[tw] OR “Brief Early Skills and Support Index”[tw] OR “Picture Arrangement Test-Adapted Version”[tw] OR “Developmental Vocabulary Assessment for Parents”[tw] OR “Reading Strategy Performance Tests”[tw] OR “Functional Literacy Experience Scale based upon Ecological Theory”[tw] OR “Preschool Screening Measure”[tw] OR “Early Motor Questionnaire”[tw] OR “Comprehensive Test of Phonological Processing–Second Edition”[tw] OR “Letter Names Measure”[tw] OR “Brief Reading Rating Scale”[tw] OR “Brief Infant-Toddler Social and Emotional Assessment-Dutch Version”[tw] OR “Infant Toddler Home Observation for Measurement of the Environment-Adapted Version”[tw] OR “Semantic Verbal Fluency Measure”[tw] OR “P1 Reading Assessments”[tw] OR “Reading Rating Form”[tw] OR “Colorado Learning Difficulties Questionnaire”[tw] OR “Reading Comprehension Test for Turkish Students”[tw] OR “Early Childhood Screening Assessment”[tw] OR “Woodcock-Johnson III Tests of Cognitive Abilities-Brazilian Version”[tiab] OR “Screening Instrument for 3-Year-Olds”[tw] OR “BRIGANCE Comprehensive Inventory of Basic Skills II”[tw] OR “Lucknow Development Screen”[tiab] OR “Screening Test for the Early Prediction of School Success”[tw] OR “Screening Test for the Early Prediction of School Success”[tw] OR “Capute Scales”[tw] OR “Middle-Childhood Home Observation for Measurement of the Environment Subscales”[tw] OR “Merrill-Palmer-Revised Scales of Development”[tiab] OR “Woodcock Diagnostic Reading Battery”[tiab] OR “Brief Academic Competence Evaluation Screening System”[tw] OR “Kindergarten Behavior Rating Scale”[tw] OR “Child Evaluation Measure”[tw] OR “Stoelting Brief Intelligence Test”[tiab] OR “Cassle-DeMoulin Child Development Age Scale”[tiab] OR “Icelandic Parent Report Checklist”[tw] OR “Infant-Toddler Development Assessment”[tw] OR “Icelandic Parent Development Inventory”[tw] OR “Computerized Neurocognitive Battery”[tw] OR “Child Development Inventory”[tw] OR “Parent Report Instrument”[tw] OR “Home Observation Measurement of the Environment Inventory-Elementary Version”[tw] OR “Teacher's Report Form”[tw] OR “Home Environment Questionnaire”[tw] OR “Preschool Rating Scale for Teachers”[tw] OR “Hayes Early Identification Listening Response Test”[tiab] OR “Preschool Screener”[tw] OR “Utrecht”[tiab] OR “Early Arithmetic, Reading and Learning”[tw] OR “Early Arithmetic Reading and Learning”[tw] OR “Mathematical Ability Instrument*”[tw] OR “Early Mathematics Assessment*”[tw] OR “iPad”[tiab] OR “iPad Early Mathematics Assessment*”[tw] “Nine-Card”[tw] OR “MindStreams”[tw] OR “Mind Streams”[tw] OR “tempe”[tiab] OR “Head-Toes-Knees-Shoulders”[tw] OR “Executive Function Scale”[tw] OR “Wisconsin Card”[tw] OR “Behavioral Rating Inventory”[tw] OR “Child Behavior Rating Scale”[tw] OR “Hayling”[tw] OR “Attention and Impulse”[tw] “Emotional Regulation Checklist”[tw] OR "Dimensional Change Card Sort"[tiab] OR "Family Life Project"[tiab] OR “Differential Abilities Scale*”[tw] OR “Batelle Development Inventory”[tw] OR “Rapid Neurodevelopment Assessment*”[tw] OR “RNDA”[tw] OR “Kilifi Developmental Inventory”[tw] OR “Kilifi Communicative Development Inventories”[tw] OR “KDI”[tw] OR “Malawi Developmental Assessment Tool”[tw] OR “MDAT”[tw] OR “Ten Question Questionnaire*”[tw] OR “Ten Questions Questionnaire*”[tw] OR “International Guide for Monitoring Child Development”[tw] OR "Griffiths Mental Development Scales"[tw] OR GMDS[tw] OR (Griffiths[tiab] AND scale*[tw]) OR "Wechsler Preschool"[tw] OR (wecshler[tw] AND scale*[tw]) OR "Stanford Binet"[tw] OR "Bracken Basic Concept Scale"[tw] OR "Peabody Picture Vocabulary Test"[tw]

OR "Expressive Vocabulary Test"[tw] OR "Preschool Language Scale*"[tw] OR "Receptive One-Word Picture Vocabulary Test*"[tw] OR "Expressive One-Word Picture Vocabulary Test*"[tw] OR "Child Behavior Checklist"[tw] OR "CBCL"[tw] OR "Behavior Assessment System"[tw] OR "BASC"[tw] OR (Conners Comprehensive Behaviour Rating Scale*) OR (Conners Comprehensive Behavior Rating Scale*) OR (conners[tiab] AND rating[tiab] scale*[tiab]) OR CBRS[tw] OR "Strengths and Difficulties Questionnaire"[tw] OR SDQ[tw] OR (vanderbilt[tiab] AND assessment[tiab] AND scale*) OR (nichq AND (scale* OR assessment)) OR "Behavior Rating Inventory of Executive Function"[tw] OR (Developmental[tiab] AND neuropsychological[tiab] AND Assessment[tiab]) OR NEPSY[tw] OR "Bracken School Readiness Assessment"[tw] OR (bracken[tiab] NOT (fern[tw] OR Pteridium[tw] OR bracken[au])) OR "BSRA"[tw] OR "Bracken Basic Concept Scale"[tw] OR BBCS[tw] OR (abc[tiab] AND inventor*[tiab]) OR "Ages and Stages Questionnaire"[tw] OR ASQ[tw] OR ("ages and stages"[tw] AND questionnaire*[tw]) OR "EASQ"[tw] OR "Extended ASQ"[tw] OR "Parents Evaluation of Developmental Status"[tw] OR (PEDS[tw] AND development[tw]) OR ((“developmental status”[tw] OR “developmental milestones”[tw]) AND parents[tw] AND evaluation[tw]) OR "PEDS:DM"[tw] OR (Denver[tiab] AND development*[tw]) OR "Beery-Buktenica Developmental Test of Visual Motor Integration"[tw] OR "Beery-Buktenica"[tw] OR "Beery VMI"[tw] OR "Bayley Scales"[tw] OR "Bayley Scale"[tw] OR "Bayley III"[tw] OR "MacArthur-Bates Communicative Development Inventories"[tw] OR "MacArthur-Bates Communicative Development Inventory"[tw] OR (MacArthur[tiab] AND CDI[tiab]) OR "MB CDI"[tw] OR (CDI[tiab] AND words[tiab] AND (gestures[tiab] OR sentences[tiab])) OR "CDI-III"[tw] OR (("Communicative Development Inventories"[tw] OR "Communicative Development Inventory"[tw] OR "CDI"[tw]) AND (Words[tw] OR Gestures[tw] OR Sentences[tw])) OR "CDI:WG"[tw] OR "CDI:WS"[tw] OR (wechsler[tiab] AND intelligence[tiab] AND scale*) OR "WISC"[tw]) AND (income[MeSH Terms] OR social class[MeSH Terms] OR employment[MeSH Terms] OR educational measurement[MeSH Terms] OR ((income[tiab] OR employment[tiab] OR “academic achievement*”[tiab] OR “educational achievement*”[tiab] OR “educational measurement”[tiab] OR “school performance”[tiab] OR “social class”[tiab]) AND 2017:2018[pdat] NOT medline[sb]))) AND (predict*[tiab] OR estima*[tiab] OR associat*[tiab]) AND (child[MeSH Terms] OR adolescent[MeSH Terms] OR infant[MeSH Terms] OR child[tw] OR children[tw] OR adolescen*[tw] OR juvenile*[tw] OR infant[tw] OR infants[tw] OR youth[tw] OR youths[tw] OR teens[tw] OR teenage*[tw] OR preschooler*[tw]) AND (English[la] or French[la]) AND (("1990/01/01"[PDat] : "3000/12/31"[PDat])) NOT (Letter[pt] OR Editorial[pt] OR Review[pt] OR News[pt] OR Meta-Analysis[pt] OR Guideline[pt] OR Cross-Sectional Studies[MeSH Terms] OR Seroepidemiologic Studies[MeSH Terms] OR Controlled Before-After Studies[MeSH Terms])

PSYCINFO SEARCH

(AB “Swedish Communicative Development Inventory III” OR AB “Kindergarten Student Entrance Profile-Adapted Version” OR AB “Environmental Symbols Instrument” OR AB “Representational Knowledge Assessment” OR AB “SEEK Observational Instrument” or AB “Nonword Reading Task” OR AB “Kindergarten Readiness Checklist-Chinese Version” OR AB “Object-based Pattern Reasoning Assessment” OR AB “Australian English Communicative Development Inventory” OR AB “Child Performance Skills Questionnaire-Adapted” OR AB “Brief Early Skills and Support Index” OR AB “Picture Arrangement Test-Adapted Version” OR AB “Developmental Vocabulary Assessment for Parents” OR AB “Reading Strategy Performance Tests” OR AB “Functional Literacy Experience Scale based upon Ecological Theory” OR AB “Preschool Screening Measure” OR AB “Early Motor Questionnaire” OR AB “Comprehensive Test of Phonological Processing–Second Edition” OR AB “Letter Names Measure” OR AB “Brief Reading Rating Scale” OR AB “Brief Infant-Toddler Social and Emotional Assessment-Dutch Version” OR AB “Infant Toddler Home Observation for Measurement of the

Environment-Adapted Version” OR AB “Semantic Verbal Fluency Measure” OR AB “P1 Reading Assessments” OR AB “Reading Rating Form” OR AB “Colorado Learning Difficulties Questionnaire” OR AB “Reading Comprehension Test for Turkish Students” OR AB “Early Childhood Screening Assessment” OR AB “Woodcock-Johnson III Tests of Cognitive Abilities-Brazilian Version” OR AB “Screening Instrument for 3-Year-Olds”OR AB “BRIGANCE Comprehensive Inventory of Basic Skills II” OR AB “Lucknow Development Screen” OR AB “Screening Test for the Early Prediction of School Success” OR AB “Screening Test for the Early Prediction of School Success” OR AB “Capute Scales” OR AB “Middle-Childhood Home Observation for Measurement of the Environment Subscales” OR AB “Merrill-Palmer-Revised Scales of Development” OR AB “Woodcock Diagnostic Reading Battery” OR AB “Brief Academic Competence Evaluation Screening System” OR AB “Kindergarten Behavior Rating Scale” OR AB “Child Evaluation Measure” OR AB “Stoelting Brief Intelligence Test” OR AB “Cassle-DeMoulin Child Development Age Scale” OR AB “Icelandic Parent Report Checklist” OR AB “Infant-Toddler Development Assessment” OR AB “Icelandic Parent Development Inventory” OR AB “Computerized Neurocognitive Battery” OR AB “Child Development Inventory” OR AB “Parent Report Instrument” OR AB “Home Observation Measurement of the Environment Inventory-Elementary Version” OR AB “Teacher's Report Form” OR AB “Home Environment Questionnaire” OR AB “Preschool Rating Scale for Teachers” OR AB “Hayes Early Identification Listening Response Test” OR AB “Preschool Screener” OR AB “Utrecht” OR AB “Early Arithmetic, Reading and Learning” OR AB “Early Arithmetic Reading and Learning” OR AB “Mathematical Ability Instrument*” OR AB “Early Mathematics Assessment*” OR AB “iPad” OR AB “iPad Early Mathematics Assessment*” OR AB “Nine-Card” OR AB “MindStreams” OR AB “Mind Streams” OR AB “tempe” OR AB “Head-Toes-Knees-Shoulders” OR AB “Executive Function Scale” OR AB “Wisconsin Card” OR AB “Behavioral Rating Inventory” OR AB “Child Behavior Rating Scale” OR AB “Hayling” OR AB “Attention and Impulse” OR AB “Emotional Regulation Checklist” OR AB "Dimensional Change Card Sort" OR AB "Family Life Project" OR AB “Differential Abilities Scale*” OR AB “Batelle Development Inventory” OR AB “Rapid Neurodevelopment Assessment*” OR AB “RNDA” OR AB “Kilifi Developmental Inventory” OR AB “Kilifi Communicative Development Inventories” OR AB “KDI” OR AB “Malawi Developmental Assessment Tool” OR AB “MDAT” OR AB “Ten Question Questionnaire*” OR AB “Ten Questions Questionnaire*” OR AB “International Guide for Monitoring Child Development” OR AB "Griffiths Mental Development Scales" OR AB GMDS OR (AB Griffiths AND AB scale*) OR AB "Wechsler Preschool" OR (AB weschler AND AB scale*) OR AB "Stanford Binet" OR AB "Bracken Basic Concept Scale" OR AB "Peabody Picture Vocabulary Test" OR AB "Expressive Vocabulary Test" OR AB "Preschool Language Scale*" OR AB "Receptive One-Word Picture Vocabulary Test*" OR AB "Expressive One-Word Picture Vocabulary Test*" OR AB "Child Behavior Checklist" OR AB "CBCL" OR AB "Behavior Assessment System" OR AB "BASC" OR (Conners Comprehensive Behaviour Rating Scale*) OR (Conners Comprehensive Behavior Rating Scale*) OR (AB conners AND AB rating AND AB scale*) OR AB CBRS OR AB "Strengths and Difficulties Questionnaire" OR AB SDQ OR (AB vanderbilt AND AB assessment AND scale*) OR (nichq AND (scale* OR assessment)) OR AB "Behavior Rating Inventory of Executive Function" OR (AB Developmental AND AB neuropsychological AND AB Assessment) OR AB NEPSY OR AB "Bracken School Readiness Assessment" OR (AB bracken NOT (AB fern OR AB Pteridium OR AB bracken)) OR AB "BSRA" OR AB "Bracken Basic Concept Scale" OR AB BBCS OR (AB abc AND AB inventor*) OR AB "Ages and Stages Questionnaire" OR AB ASQ OR ("ages and stages" AND questionnaire*) OR AB "EASQ" OR AB "Parents Evaluation of Developmental Status" OR (AB PEDS AND AB development) OR ((AB “developmental status” OR AB “developmental milestones”) AND AB parents AND AB evaluation) OR AB "PEDS:DM" OR (AB Denver AND AB development*) OR AB "Beery-Buktenica Developmental Test of Visual Motor Integration" OR AB "Beery-Buktenica" OR AB "Beery VMI" OR AB "Bayley Scales" OR AB "Bayley Scale" OR AB "Bayley III" OR AB "MacArthur-Bates Communicative Development Inventories" OR AB "MacArthur-Bates Communicative Development

Inventory" OR (AB MacArthur AND AB CDI) OR AB "MB CDI" OR (AB CDI AND AB words AND (AB gestures OR AB sentences)) OR AB "CDI-III" OR ((AB "Communicative Development Inventories" OR AB "Communicative Development Inventory" OR AB "CDI") AND (AB Words OR AB Gestures OR AB Sentences)) OR AB "CDI:WG" OR AB "CDI:WS" OR (AB wechsler AND AB intelligence AND scale*) OR AB "WISC") AND (DE "Income Level" OR DE "Salaries" OR DE "Socioeconomic Status" OR DE "Poverty" OR DE "Employment Status" OR "Social Class" OR DE "Educational Attainment Level" OR DE "Academic Achievement" OR "Academic Achievement Prediction") AND (AB predict* OR AB estima* OR AB associat* OR Statistical Validity) AND (DE "Adolescents" OR DE "Children" OR DE "Early Adolescents" OR DE "Late Adolescents" OR DE "Youth" OR AB "children" OR AB adolescen* or AB juvenile* or AB infant* OR AB youth* OR AB "teens" OR AB teenage* OR AB "Preschool Students") AND (LA English OR LA French) AND DT 1990-2018 AND (PT "Peer Reviewed Journal") NOT ("cross-sectional" OR "controlled before-after stud*" OR "population survey" OR "population-based survey")

ERIC SEARCH

(AB "Swedish Communicative Development Inventory III" OR AB "Kindergarten Student Entrance Profile-Adapted Version" OR AB "Environmental Symbols Instrument" OR AB "Representational Knowledge Assessment" OR AB "SEEK Observational Instrument" OR AB "Nonword Reading Task" OR AB "Kindergarten Readiness Checklist-Chinese Version" OR AB "Object-based Pattern Reasoning Assessment" OR AB "Australian English Communicative Development Inventory" OR AB "Child Performance Skills Questionnaire-Adapted" OR AB "Brief Early Skills and Support Index" OR AB "Picture Arrangement Test-Adapted Version" OR AB "Developmental Vocabulary Assessment for Parents" OR AB "Reading Strategy Performance Tests" OR AB "Functional Literacy Experience Scale based upon Ecological Theory" OR AB "Preschool Screening Measure" OR AB "Early Motor Questionnaire" OR AB "Comprehensive Test of Phonological Processing–Second Edition" OR AB "Letter Names Measure" OR AB "Brief Reading Rating Scale" OR AB "Brief Infant-Toddler Social and Emotional Assessment-Dutch Version" OR AB "Infant Toddler Home Observation for Measurement of the Environment-Adapted Version" OR AB "Semantic Verbal Fluency Measure" OR AB "P1 Reading Assessments" OR AB "Reading Rating Form" OR AB "Colorado Learning Difficulties Questionnaire" OR AB "Reading Comprehension Test for Turkish Students" OR AB "Early Childhood Screening Assessment" OR AB "Woodcock-Johnson III Tests of Cognitive Abilities-Brazilian Version" OR AB "Screening Instrument for 3-Year-Olds" OR AB "BRIGANCE" OR AB "Lucknow Development Screen" OR AB "Screening Test for the Early Prediction of School Success" OR AB "Screening Test for the Early Prediction of School Success" OR AB "Capute Scales" OR AB "Middle-Childhood Home Observation for Measurement of the Environment Subscales" OR AB "Merrill-Palmer-Revised Scales of Development" OR AB "Woodcock Diagnostic Reading Battery" OR AB "Brief Academic Competence Evaluation Screening System" OR AB "Kindergarten Behavior Rating Scale" OR AB "Child Evaluation Measure" OR AB "Stoelting Brief Intelligence Test" OR AB "Cassle-DeMoulin Child Development Age Scale" OR AB "Icelandic Parent Report Checklist" OR AB "Infant-Toddler Development Assessment" OR AB "Icelandic Parent Development Inventory" OR AB "Computerized Neurocognitive Battery" OR AB "Child Development Inventory" OR AB "Parent Report Instrument" OR AB "Home Observation Measurement of the Environment Inventory-Elementary Version" OR AB "Teacher's Report Form" OR AB "Home Environment Questionnaire" OR AB "Preschool Rating Scale for Teachers" OR AB "Hayes Early Identification Listening Response Test" OR AB "Preschool Screener" OR AB "Utrecht" OR AB "Early Arithmetic, Reading and Learning" OR AB "Early Arithmetic Reading and Learning" OR AB "Mathematical Ability Instrument*" OR AB "Early Mathematics Assessment*" OR AB "iPad" OR AB "iPad Early Mathematics Assessment*" OR AB "Nine-Card" OR AB "MindStreams" OR AB "Mind Streams" OR AB "tempe" OR AB "Head-Toes-Knees-Shoulders" OR AB "Executive Function Scale" OR

AB “Wisconsin Card” OR AB “Behavioral Rating Inventory” OR AB “Child Behavior Rating Scale” OR AB “Hayling” OR AB “Attention and Impulse” OR AB “Emotional Regulation Checklist” OR AB "Dimensional Change Card Sort" OR AB "Family Life Project" OR AB “Differential Abilities Scale*” OR AB “Batelle Development Inventory” OR AB “Rapid Neurodevelopment Assessment*” OR AB “RNDA” OR AB “Kilifi Developmental Inventory” OR AB “Kilifi Communicative Development Inventories” OR AB “KDI” OR AB “Malawi Developmental Assessment Tool” OR AB “MDAT” OR AB “Ten Question Questionnaire*” OR AB “Ten Questions Questionnaire*” OR AB “International Guide for Monitoring Child Development” OR AB "Griffiths Mental Development Scales" OR AB GMDS OR (AB Griffiths AND AB scale*) OR AB "Wechsler Preschool" OR (AB weschler AND AB scale*) OR AB "Stanford Binet" OR AB "Bracken Basic Concept Scale" OR AB "Peabody Picture Vocabulary Test" OR AB "Expressive Vocabulary Test" OR AB "Preschool Language Scale*" OR AB "Receptive One-Word Picture Vocabulary Test*" OR AB "Expressive One-Word Picture Vocabulary Test*" OR AB "Child Behavior Checklist" OR AB "CBCL" OR AB "Behavior Assessment System" OR AB "BASC" OR (Conners Comprehensive Behaviour Rating Scale*) OR (Conners Comprehensive Behavior Rating Scale*) OR (AB conners AND AB rating AND AB scale*) OR AB CBRS OR AB "Strengths and Difficulties Questionnaire" OR AB SDQ OR (AB vanderbilt AND AB assessment AND scale*) OR (nichq AND (scale* OR assessment)) OR AB "Behavior Rating Inventory of Executive Function" OR (AB Developmental AND AB neuropsychological AND AB Assessment) OR AB NEPSY OR AB "Bracken School Readiness Assessment" OR (AB bracken NOT (AB fern OR AB Pteridium OR AB bracken)) OR AB "BSRA" OR AB "Bracken Basic Concept Scale" OR AB BBCS OR (AB abc AND AB inventor*) OR AB "Ages and Stages Questionnaire" OR AB ASQ OR ("ages and stages" AND questionnaire*) OR AB "EASQ" OR AB "Parents Evaluation of Developmental Status" OR (AB PEDS AND AB development) OR ((AB “developmental status” OR AB “developmental milestones”) AND AB parents AND AB evaluation) OR AB "PEDS:DM" OR (AB Denver AND AB development*) OR AB "Beery-Buktenica Developmental Test of Visual Motor Integration" OR AB "Beery-Buktenica" OR AB "Beery VMI" OR AB "Bayley Scales" OR AB "Bayley Scale" OR AB "Bayley III" OR AB "MacArthur-Bates Communicative Development Inventories" OR AB "MacArthur-Bates Communicative Development Inventory" OR (AB MacArthur AND AB CDI) OR AB "MB CDI" OR (AB CDI AND AB words AND (AB gestures OR AB sentences)) OR AB "CDI-III" OR ((AB "Communicative Development Inventories" OR AB "Communicative Development Inventory" OR AB "CDI") AND (AB Words OR AB Gestures OR AB Sentences)) OR AB "CDI:WG" OR AB "CDI:WS" OR (AB wechsler AND AB intelligence AND scale*) OR AB "WISC") AND (DE "Income" OR DE "Salaries" OR DE "Wages" OR DE "Economic Status" OR DE "Poverty" OR DE "Employment Level" OR "Social Class" OR DE "Socioeconomic Status" OR DE "Educational Attainment" OR DE "Academic Achievement" OR DE "Educational Attainment") AND (AB predict* OR AB estima* OR AB associat*) AND (DE "Adolescents" OR DE "Children" OR DE "Early Adolescents" OR DE "Late Adolescents" OR DE "Youth" OR AB children OR AB adolescen* or AB juvenile* or AB infant OR AB infants OR AB youth OR AB youths OR AB teens OR AB teenage* OR AB preschooler*) AND (LA English OR LA French) AND DT 1990-2018 NOT (“cross-sectional” OR “controlled before-after stud*” OR “population survey” OR “population-based survey”)

SUPPLEMENTAL TABLE 1. Child development assessment tools included in search string

1. Ages and Stages Questionnaire	28. Computerized Neurocognitive Battery
2. Australian English Communicative Development Inventory	29. Conners Comprehensive Behaviour Rating Scale
3. Batelle Development Inventory	30. Denver Developmental Screening Test
4. Bayley Scales of Infant and Toddler Development	31. Denver II
5. Beery-Buktenica Developmental Test of Visual Motor Integration	32. Developmental NEuroPSYchological Assessment (NEPSY)
6. Behavior Assessment System for Children (BASC)	33. Developmental Vocabulary Assessment for Parents
7. Behavior Rating Inventory of Executive Function (BRIEF)	34. Differential Abilities Scale
8. Bracken Basic Concept Scale	35. Dimensional Change Card Sort
9. Bracken School Readiness Assessment	36. Early Arithmetic Reading and Learning
10. Brief Academic Competence Evaluation Screening System	37. Early Childhood Screening Assessment
11. Brief Early Skills and Support Index	38. Early Mathematics Assessment
12. Brief Infant-Toddler Social and Emotional Assessment	39. Early Motor Questionnaire
13. Brief Reading Rating Scale	40. Emotional Regulation Checklist
14. BRIGANCE Comprehensive Inventory of Basic Skills II	41. Environmental Symbols Instrument
15. Capute Scales	42. Executive Function Scale
16. Cassle-DeMoulin Child Development Age Scale	43. Expressive One-Word Picture Vocabulary Test
17. Child Behavior Checklist – Parent-Report Form	44. Expressive Vocabulary Test
18. Child Behavior Checklist – Teacher-Report Form	45. Extended Ages and Stages Questionnaire
19. Child Behavior Checklist – Youth Self-Report	46. Family Life Project
20. Child Behavior Rating Scale	47. Functional Literacy Experience Scale based upon Ecological Theory
21. Child Development Inventory	48. Griffiths Mental Development Scales
22. Child Evaluation Measure	49. Hayes Early Identification Listening Response Test
23. Child Hayling Test	50. Head-Toes-Knees-Shoulders
24. Child Performance Skills Questionnaire-Adapted	51. Home Environment Questionnaire
25. Colorado Learning Difficulties Questionnaire	52. Home Observation Measurement of the Environment Inventory-Elementary Version
26. Communicative Development Inventories	53. Icelandic Parent Development Inventory
27. Comprehensive Test of Phonological Processing–Second Edition	54. Icelandic Parent Report Checklist
	55. Infant Toddler Home Observation for Measurement of the Environment-Adapted Version Semantic Verbal Fluency Measure
	56. Infant-Toddler Development Assessment
	57. International Guide for Monitoring Child Development

58. Kilifi Communicative Development Inventories
59. Kindergarten Behavior Rating Scale
60. Kindergarten Readiness Checklist
61. Kindergarten Student Entrance Profile-Adapted Version
62. Letter Names Measure
63. Lucknow Development Screen
64. MacArthur-Bates Communicative Development Inventories
65. Malawi Developmental Assessment Tool
66. Mathematical Ability Instrument
67. Merrill-Palmer-Revised Scales of Development
68. Middle-Childhood Home Observation for Measurement of the Environment
69. MindStreams Expanded Go-NoGo Response Inhibition
70. Mullen Scales of Early Learning
71. NICHQ Vanderbilt Assessment Scales
72. Nine-Card Sorting Test
73. Nonword Reading Task
74. Object-based Pattern Reasoning Assessment
75. P1 Reading Assessments
76. Parent Report Instrument
77. Parents Evaluation of Developmental Status
78. Peabody Picture Vocabulary Test
79. Picture Arrangement Test-Adapted Version
80. Preschool Language Scale
81. Preschool Rating Scale for Teachers
82. Preschool Screener
83. Preschool Screening Measure
84. Rapid Neurodevelopment Assessment
85. Reading Comprehension Test for Turkish Students
86. Reading Rating Form
87. Reading Strategy Performance Tests
88. Receptive One-Word Picture Vocabulary Test

89. Representational Knowledge Assessment
90. Screening Instrument for 3-Year-Olds
91. Screening Test for the Early Prediction of School Success
92. SEEK Observational Instrument
93. Stanford-Binet Intelligence Scales
94. Stoelting Brief Intelligence Test
95. Strengths and Difficulties Questionnaire
96. Swedish Communicative Development Inventory III
97. Teacher-Rated Children's Attention and Impulse Control Questionnaire
98. Ten Question Questionnaire
99. Utrecht Early Mathematical Competence Scales
100. Wechsler Intelligence Scale for Children (WISC)
101. Wechsler Preschool & Primary Scale of Intelligence (WPPSI)
102. Wisconsin Card Sorting Test
103. Woodcock Diagnostic Reading Battery
104. Woodcock-Johnson III Tests of Cognitive Abilities

SUPPLEMENTAL TABLE 2. Details of studies included in the review

**Reference** Study design & population	**Sample size**	**Assessment tool**	**Assessment age**	**Outcome Measure**	**Outcome age**	**Duration of follow-up**	**Effect measure**
**Clarren, 1993** Retrospective cohort, Pacific Northwest of U.S.	Kindergarten: 102	Wechsler Intelligence Scales for Children (WISC) Verbal and Performance IQ	5 years	**Achievement:** Standardized Achievement Tests	14-15 years	10 years	IQ model regression estimate for total achievement in Grade 9: 0.56**
		Reitan-Indiana Neuropsychological Battery for Children					Neuropsychological model regression estimate for total achievement in Grade 9: 0.63**
	2nd grade: 113	Wechsler Intelligence Scales for Children (WISC) Verbal and Performance IQ	8 years	**Achievement:** Standardized Achievement Tests	14-15 years	8 years	IQ model regression estimate for total achievement in Grade 9: 0.51*
		Reitan-Indiana Neuropsychological Battery for Children					Neuropsychological model regression estimate for total achievement in Grade 9: 0.63**
**Richards 1995** Prospective cohort, Children with learning disabilities in a private residential school in	20	Child Behavior Checklist, Children's Assessment and Adjustment Survey	11-17 years	**Achievement:** WRAT-R Reading, Spelling, and Math Scores	11-17 years	1-2 years	Teacher report of externalizing behavior:<br>Reading regression coefficient: -0.57*<br>Spelling regression coefficient: - 0.55**<br>Math regression coefficient:<br>-0.33 N.S.<br><br>Parent report of externalizing behavior:<br>Reading regression coefficient: -0.26 N.S.<br>Spelling regression coefficient: 0.05 N.S.<br>Math regression coefficient: -0.28 N.S.

Atlantic Canada							
**Lamp 2001** Prospective cohort, Children from impoverished families in the U.S.	Phase 1: 67	Stanford-Binet Intelligence Scale: LM (SB:LM)	4 years	**Achievement:** Metropolitan Achievement Test	5 - 10 years	1-5 years	Correlation coefficient kindergarten to 6th grade: 0.39**
	Phase 1: 67<br>Phase 2: 59	Stanford-Binet Intelligence Scale: Fourth Edition (SB:FE)	4, 6, 9 years				Correlation coefficient kindergarten to 6th grade: 0.47**<br><br>Correlation 1st to 6th grade: 0.52**<br><br>Correlation 4th to 6th grade: 0.62**
**Fergusson 2005** Retrospective cohort, Participants of Christchurch Child Development Study in New Zealand	1265	Wechsler Intelligence Scales for Children-Revised	8-9 years	**Wealth:** Gross income ($) during past 12 months & rates of unemployment (18-25 years)	18-25 years	10-17 years	Regression coefficient gross income: 1.595*<br><br>Regression coefficient duration of unemployment: -0.14*
				**Attainment:** Educational Outcomes (18 years) & gained university degree (25 years)			Regression coefficient gained school qualifications: .82**<br><br>Regression coefficient gained university degree: 0.67 **

**Rothon 2009** Prospective cohort, School-based cohort in East London	1718	Strengths and Difficulties Questionnaire (SDQ)	13-14 years	**Achievement:** General Certificate of Secondary Education Scores	15-16 years	2 years	<u>Odds of receiving 5 or more A*-C grades given psychological distress:</u><br>OR boys: 0.41**<br>OR girls: 0.60**
		Short Moods and Feelings Questionnaire (SMFQ)					<u>Odds of receiving 5 or more A*-C grades given psychological distress:</u><br>OR boys: 0.58**<br>OR girls: 0.83 N.S.
**Moffitt 2011** Prospective cohort, Dunedin Multidisciplinary Health and Development Study cohort in New Zealand	1037	Wechsler Intelligence Scales for Children-Revised	7,9,11 years averaged and standardized scores	**Wealth:** SES	32 years	21-29 years	SES regression estimate: -0.400*
				**Wealth:** Income			Income regression estimate: -0.291*
				**Wealth:** Financial issues			Financial planfulness regression estimate: -0.160*<br><br>Financial struggles regression estimate: 0.029*
**McClelland 2013** Prospective cohort, Colorado Adoption Project cohort	286	Peabody Picture Vocabulary Test	4 years	**Attainment:** College completion age 25	21 or 25 years	>17 years	OR college completion age 25 regression estimate: 1.03 (N.S.)
		Peabody Individual Achievement Test-Reading subscale	7 years	**Attainment:** College completion age 25			College completion age 25 correlation coefficient: 0.16*

		Wechsler Intelligence Scales for Children-Revised Arithmetic subscale	7 years old	**Attainment:** college completion age 25			College completion age 25 correlation coefficient: 0.13*
**Sagatun 2014** Retrospective cohort, Students in academic and vocational schools from Norwegian registry	10,931	Strengths and Difficulties Questionnaire	15-16 years	**Attainment:** National registry of school completion	20-21 years	5 years	<u>Odds of non-completion given externalizing problems:</u><br>OR boys: 1.38***<br>OR: 1.48***<br><br><u>Odds of non- completion given internalizing problems:</u><br>OR boys: 1.11***<br>OR girls: 1.21***
**Veldman 2014** Prospective cohort, Dutch cohort from the Tracking Adolescents' Individual Lives Survey	1,711	Youth Self Report & Child Behavior Checklist (Combined)	15-16 years	**Attainment:** Self-report of years of schooling completed	19 years	8 years	<u>Odds of low vs. medium educational attainment:</u><br>OR externalizing problems: 1.70**<br>OR internalizing problems: 1.32 N.S.<br>OR attention problems: 1.78**<br><br><u>Odds of medium vs. high educational attainment:</u><br>OR externalizing problems: 1.56**<br>OR internalizing problems 1.25 NS<br>OR attention problems: 1.53**<br><br>*Note: low (primary, lower vocational and lower secondary education), medium (intermediate vocational and intermediate secondary), and high (higher secondary, higher vocational and university)*

**Samuels 2016** Prospective cohort, Urban charter school in New York City	322	Behavior Rating Inventory of Executive Function (BRIEF) By Teacher and Teacher's Assistant	12-15 years	**Achievement:** School grade point averages	12-15 years	4 years	<u>Teacher report:</u><br>English and language arts regression coefficient: -0.47*<br>Math regression coefficient: -0.53*<br>Science regression coefficient: -0.50 N.S.<br>Social studies regression coefficient: 0.56*<br>Spanish regression coefficient: -0.41*<br><br><u>Teacher's assistant report:</u><br>English and language arts regression coefficient: -0.33*<br>Math regression coefficient: -0.47*<br>Science regression coefficient: -0.59 N.S.<br>Social studies regression coefficient: -0.52*<br>Spanish regression coefficient: -0.43*
**Gygi 2017** Prospective cohort, Elementary school children in German-speaking Switzerland	103	Wechsler Intelligence Scale for Children - Fourth Edition	6 - 11 years	**Achievement:** Self-reported school grades	9 -14 years	3 years	Average school grade regression estimate: 0.128**<br><br>Math school grades regression estimate: 0.242 (N.S.)<br><br>Language school grades regression estimate: 0.260**<br><br>*Adjustment for sex and age*
OR odds ratio; N.S. Not statistically significant at p <0.05; * p<0.05; ** p<0.01; *** p<0.001							
